# Determinants of Well-Being: A Causal Framework

**DOI:** 10.1007/s11205-026-03816-w

**Published:** 2026-03-17

**Authors:** Lea A. Tamberg, Vivien Fisch-Romito, Julia K. Steinberger

**Affiliations:** Institute of Geography and Sustainability, University of Lausanne, Lausanne, Switzerland

**Keywords:** Well-being, Causality, Eudaimonic, Hedonic, Sustainability, Subjective

## Abstract

Different philosophical strands have developed distinct well-being conceptions, which are nevertheless strongly linked. For instance, many typical well-being components in eudaimonic philosophies correlate with subjective life assessment measures. Existing empirical research also provides many insights into which factors determine human well-being, such as health and the quality of relationships. However, the causal ordering of these factors is often not explicitly considered, although doing so would be crucial for the identification of intervention points and a correct estimation of effect sizes in empirical studies. In response to these two observations, we propose a unified causal framework of the main pathways determining human well-being in its different conceptions, with a focus on those prominent in sustainability research and the subjective well-being community. The framework considers environmental, economic, societal, political, social, and psychological factors and shows how they interact. It combines insights from theories of human needs, the capabilities approach, and research on subjective indicators. We illustrate the use of the framework with examples of societal interventions on well-being, environmental impacts, and sustainable well-being policies.

## Introduction

Many scientific disciplines work on a better understanding of human well-being and its determinants. In recent decades, research on subjective well-being (SWB) – how people experience their lives affectively and cognitively (Diener, 1984) – has produced substantial empirical advances on the predictors of different well-being measures (Diener et al., 2018a). Well-being is also an important concept in our own area of sustainability research, which analyses how to enable human well-being while limiting ecological impact (Rogers et al., 2012; Dietz, 2023) and how ecological degradation impacts well-being (Adger et al., 2022; Schrijver et al., 2025). Sustainability research thus depends on a thorough understanding of well-being, its measurement, and its causes. The sustainability literature often refers to empirical research results from the SWB community, but is also characterised by diversity in well-being theories and assessment methods (Lamb & Steinberger, 2017; Brand-Correa & Steinberger, 2017). For instance, while SWB research is by definition only interested in subjective assessments, objective well-being indicators are common in sustainability research.

In this diverse research landscape, we make two observations.

First, although different philosophical strands have developed distinct definitions of well-being, the different conceptions have strong causal links with each other, and should be seen as components of a common complex system. We summarise the different philosophical conceptions of well-being most prominent in the SWB and the sustainability communities in section 2, while also explaining some terminological inconsistencies between these communities that might cause misunderstandings in interdisciplinary research.

Second, existing empirical research provides many insights into which factors determine human well-being, especially subjective life assessments (Veenhoven, 2010; Clark et al., 2018; Diener et al., 2018a; Deeming, 2013; Huppert, 2009; Dolan et al., 2008; Helliwell et al., 2015). However, the causal ordering of these determinants is generally not explicitly considered. Instead, they are often presented as lists, which is not incorrect, but incomplete. To give a small example, consider the variables ‘gender’ and ‘time spent with family members’. Both influence well-being and are therefore determinants of well-being. At the same time, in a patriarchal society, ones gender also influences how much time one spends with family members. Part of the effect of gender on well-being can be explained by this differing time use. Therefore, important information is lost by simply listing gender and time use as determinants of well-being. In section 3, we explain why taking the causal structure of determinants into account is crucial for the identification of intervention points and a correct estimation of effect sizes in empirical studies.

Motivated by these two observations, we thus propose a unified causal framework of the main pathways that determine human well-being in its various conceptions, with a focus on those prominent in sustainability research and the SWB community. The framework considers environmental, economic, societal, political, social, and psychological determinants of well-being and shows how they interact. We explain the framework in section 4 and present several application examples regarding societal interventions, environmental impacts, and sustainable well-being in section 5.

Our article makes several contributions. Most importantly, our well-being framework goes beyond existing attempts to “sort” the complexity of different conceptions of well-being and their determinants.^1^ It further differentiates determinant categories, integrates more conceptions of well-being, and considers additional determinants and causal links. It understands the determinants of wellbeing and the different conceptions of well-being as components of a complex system driven by interactions and feedback loops. While its unit of analysis is the individual, it can also serve as a basis for developing population-level models of well-being.

In addition to this main contribution, the article is also relevant for readers interested in different approaches to modelling the causal determinants of well-being. Moreover, section 2 provides a clarification of the different uses of well-being terminology in the SWB and the sustainability literature.

## Different Philosophical Conceptions of Well-Being

Different philosophical schools have developed distinct conceptions of the term ‘well-being’ that can be categorized as hedonic, eudaimonic, and preferences based (Crisp, 2021; Lamb & Steinberger, 2017; Dolan et al., 2006; Diener et al., 2018b; Dodds, 1997). While for this article we take an agnostic stance on the choice of well-being conceptualisation, we here summarise the existing conceptions, as we will later see how they occupy different categories of a causal network.

### Hedonic Well-Being

According to the hedonic conception, well-being means the presence of pleasure and the absence of pain. It is based on the works of Aristippus and, in a more nuanced form, Epicure (Honderich, 2005). Epicurian hedonism has sometimes been framed as the philosophical basis for the goal of economic growth (Brand-Correa & Steinberger, 2017; Lamb & Steinberger, 2017). However, one should keep in mind that Epicure centred many of his concepts around the idea of satisfiable and unsatisfiable desires, and claimed that following the latter will always lead to unhappiness (Mitsis, 1988). Therefore, Epicure himself should be understood as an advocate for sufficiency (O’Neill, 2006) – not as an end in itself, but as a means to achieve pleasure and avoid pain in the long run. However, hedonism is insofar linked to utility maximisation (which is often used to justify growth) as hedonic well-being is the object of interest in classical utilitarianism. Utilitarianism in general is a normative ethical theory claiming that “the morally right action is the action that produces the most [overall] good” (Driver, 2022). This ‘good’ could in principle be many different things, but the “Classical Utilitarians, Jeremy Bentham and John Stuart Mill, identified the good with pleasure, so, like Epicurus, were hedonists about value” (Driver, 2022).

### Eudaimonic Well-Being

The eudaimonic conception of well-being goes back to Aristotle^2^, who claimed that a good life means living “in accordance with virtue” (Aristotle, 2014), which is often understood as living up to one’s full potential (Ryff, 1989). Eudaimonic well-being has been interpreted differently in the sustainability and SWB literature.

The sustainability literature gives little attention to the meaning of “virtue”. Instead, it identifies as the central property of eudaimonism the claim that there are objective constituents of well-being, which are ends in themselves and not just means for more positive and less negative feelings (Crisp, 2021; Haybron, 2020): “Indeed, we feel good about fostering such goods because we believe they are of value. We do not just value being in the right subjective states.” (O’Neill, 2006). This interpretation results in classifying theories of basic human needs (Doyal & Gough, 1984; Max-Neef et al., 1991) and the capabilities approach (Nussbaum, 2015; Nussbaum & Sen, 1993; Sen, 2008) as eudaimonic well-being theories (Brand-Correa & Steinberger, 2017; Lamb & Steinberger, 2017; O’Neill, 2006). Proponents of human needs theories claim that while there are infinitely many different strategies to satisfy human needs, the needs themselves are finite, few, and universal (Max-Neef et al., 1991). Moreover, the satisfaction of one need cannot substitute the satisfaction of another (Brand-Correa & Steinberger, 2017; O’Neill, 2010). The capabilities approach, in contrast, claims that it is not need satisfaction itself that counts (or, in the terminology of the capabilities approach, functionings), but what people *can* do if they want to (i.e., what capabilities they have). Nussbaum (2000) has proposed a list of “central human capabilities”, which she considers as a “moral basis of central constitutional guarantees” (Nussbaum, 2000). Despite the philosophical difference between human needs and capabilities, this list of central human capabilities can be quite straightforwardly mapped to lists of human needs (Brand-Correa et al., 2020).

In the SWB literature, well-being conceptions providing ‘objective lists’ are usually seen as a distinct category from eudaimonic well-being (Abdallah & Mahoney, 2024; Dolan et al., 2006). Instead, eudaimonia is interpreted closer to the Aristotelian ideals of virtue and fulfilment of potential, often called “flourishing”. The concrete theories differ in whether they localise flourishing in the personal traits (motivations and orientations), behaviour, or feelings of a person (Abdallah & Mahoney, 2024). The two interpretations of eudaimonia overlap insofar as many operational definitions in the SWB literature provide lists of elements very similar to human needs, especially when the definitions are focused on behaviour or functionings. Theories that explicitly use the term ‘needs’ are, for example, Self-Determination Theory (Ryan & Deci, 2000, 2001) and the theory of Humanistic Well-being (Vittersø, 2025). However, given the SWB perspective, they usually only consider subjective assessments and/or focus on “psychological” needs.

### Preference-Based Well-Being

The preference-based conception of well-being, standard in welfare economics (Crisp, 2021), refrains from defining well-being components, be it emotions or others. Instead, it proposes that the only thing that counts for the well-being of a person is the fulfilment of their individual preferences (or desires) independent of their nature. Usually (although not necessarily), this conception of well-being goes hand in hand with the assumption that, all else staying equal, more consumption is always better (Fleurbaey, 2009), although marginal returns might shrink. For a good summary of the criticisms of preference-based well-being, and especially the assumption that preferences can be revealed in a meaningful way via market decisions, see Dodds (1997). In the past, there have been attempts to reconcile preference-based conceptions of well-being with the fact that preferences can adapt to circumstances (Weizsäcker, 2024).

### Subjective and Objective Well-Being

Throughout the literature, there are inconsistent uses of the terms subjective and objective well-being. In the sustainability literature, hedonic well-being is sometimes called subjective well-being and eudaimonic well-being objective well-being (O’Neill, 2006) due to the interpretation as objective components. However, this is misleading insofar as subjective/objective can also refer to the type of assessment one might choose to determine a person’s well-being. Here, subjective means self-reported assessment, and objective means a reproducible external measurement. Well-being according to all three philosophies discussed above can be assessed both subjectively and objectively (Brand-Correa & Steinberger, 2017), as shown in Table 1,^3^. Therefore, we use ‘subjective’ and ‘objective’ for the method of assessment, rather than the underlying philosophy of well-being (Gasper, 2007).

**Table 1:**
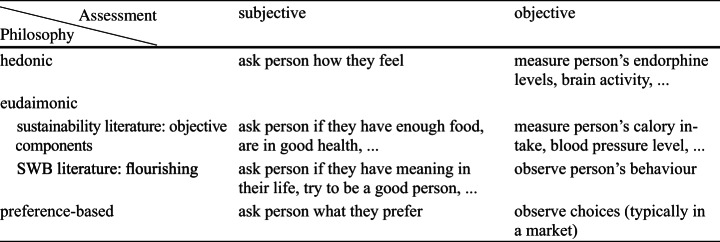
Examples for subjective and objective measurements of hedonic, eudaimonic, and preference-based well-being. Inspired by Brand-Correa & Steinberger (2017) and Gasper (2007), extended to preference-based well-being and SWB accounts of eudaimonia

In the SWB literature, the term “Subjective Well-Being” usually refers specifically to the trio of (subjectively assessed) “frequent positive affect, infrequent negative affect, and cognitive evaluations such as life satisfaction” (Diener, 1984), sometimes extended by eudaimonia (in the flourishing interpretation discussed above). Life satisfaction (or, more broadly, life assessment) is not listed in Table 1. Self-reported life assessments are obviously subjective assessments. However, their philosophical classification is controversial. Some authors classify them as hedonic (Disabato et al., 2016; Lamb & Steinberger, 2017). In contrast, Deci and Ryan (2008) point out that life satisfaction is not a strictly hedonic measure as “it involves the cognitive evaluation of the conditions of one’s life”. Similarly, O’Neill (2006) argues that respondents to life assessment surveys are more likely to try to aggregate the state of different ‘objective’ well-being constituents rather than summarise their feelings. In this logic, life assessments are subjective and aggregated measures of eudaimonic well-being in the objective components interpretation, with an unknown weighting of the different components. This weighting seems to be influenced by the exact question formulation. For instance, the Cantril Ladder question^4^ differs from the standard life satisfaction question^5^: Nilsson et al. (2024) found that the Cantril Ladder is more strongly associated with ideas of power and wealth due to the ladder motive. Independent of this question of final weighting, people could also use their personal preferences as a measure stick for different aspects of their lives. While there is initial evidence that human preferences coincide with eudaimonic well-being components (Benjamin et al., 2025), they conceptually do not have to. Due to the unclear philosophical rootedness of life assessments, some authors put them in their own category, “evaluative well-being” (Abdallah & Mahoney, 2024; Diener, 1984; Dolan et al., 2006; Layard & De Neve, 2023).

We argue that life assessments (and subjective indicators in general) are worth studying independently of the chosen well-being philosophy. They carry an important signal that can reveal potential blind spots of objective measures, regardless of how exactly people form their responses to life assessment questions. For example, in a situation of apparently good performance on objective indicators, persisting low levels of life assessment in a population could indicate that an important well-being component (or, following the hedonic perspective, an important source of pleasure or pain) has been overlooked when selecting these objective indicators.

At the same time, research on subjective indicators has shown important psychological biases that must be taken into account when interpreting the results. For instance, numerical scales used to measure life satisfaction are known to induce a simplification of the scale to “focal values”, especially among respondents with a low numeric literacy (Barrington-Leigh, 2024). Moreover, changes in objective living conditions often have only short-term effects on emotional experiences and life assessments, a phenomenon called “hedonic treadmill” (Kahneman & Krueger, 2006). From a eudaimonic perspective (in the objective components sense), subjective well-being indicators can therefore only be a complement to objective ones, although we think an important one.

## Why the Causal Structure of Well-Being Determinants Matters

Studying the determinants of well-being implies searching for factors that have a *causal effect* on well-being. The importance of causality is recognized in the literature on the determinants of well-being, for instance when stressing the difference between correlation and causation. Despite this, the field usually presents the results of well-being research in the form of lists of determinants (for examples, see Table 2 based on Veenhoven (2010); Clark et al. (2018); Diener et al. (2018a); Deeming (2013); Huppert (2009); Dolan et al. (2008); Helliwell et al. (2015)). Such a presentation ignores the fact that these determinants are inter-related, and indeed situated at different levels of an ordered causal network^6^.

**Table 2:** Determinants of Subjective Well-Being listed in some influential reviews. X signifies that this variable is presented as determinant, ^*^ signifies that variable is presented as a well-being dimension

Determinant	Huppert (2009)	Diener (2018)	Dolan et al. (2008)	Clark et al. (2018)	Helliwell (2015)	Veenhoven (2010)	Deeming (2013)
Health	*	x	x	x	x	x	x
Income	x	x	x	x	x	x	x
Socio-economic position	x					x	x
Unemployment	x	x	x	x		x	x
Genes	x	x					
Parental care	x			x			
Parent’s mental health				x			
Education				x		x	x
Community	x		x			x	
Relationship(s)	*	x	x	x	x	x	x
Age	x	x					x
Values	x				x	x	
Personality	x	x				x	
Meaningful goals	x	x					
Freedom					x	x	
Corruption		x			x		
Equality	x	x				x	
Rule of law						x	
Good citizenship						x	
Cultural pluriformity						x	
Modernity						x	
Political freedom		x					
Green space		x					

Identifying the causal structure of social systems is inherently difficult, but it is usually the ultimate motivation for researching these systems. This is because knowing the causal setup of a system enables effective *interventions* (Tay & Kuykendall, 2013), as well as a correct estimation of their effects.

**Fig. 1:**
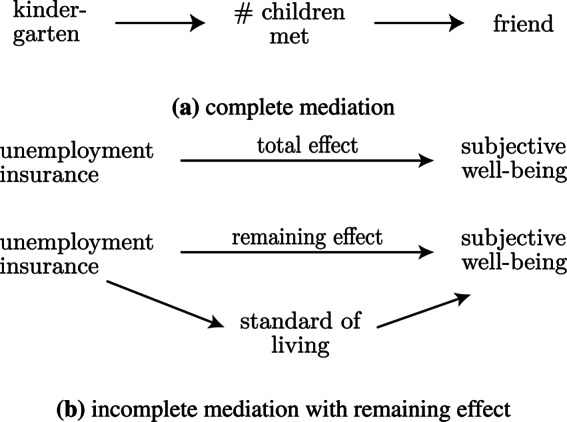
Illustrations of examples for causal mediation

The question of interventions is closely linked to the fact that causal structure carries information that goes beyond the concept of “determinants”. In colloquial terms, a variable is one of the determinants of another variable if intervening on the former (and not on any other variable) causes the latter to change. In causality theory, a term more commonly used than ‘determinant’ is ‘cause’. For a mathematically more sound definition of causal effects, see Peters et al. (2017). Here, we stick to ‘determinant’ as the term most often used in the well-being literature. The causal effect of a determinant does not have to be direct, and can be mediated via other variables, which are then also determinants. For instance, both going to kindergarten and meeting many other children make it more likely for a child to find a friend. However, the effect of going to kindergarten could be fully mediated by meeting many other children (Fig. 1a). In this case, holding constant the number of other children a child meets makes it irrelevant whether the child also goes to kindergarten or not. Simply listing both ‘kindergarten attendance’ and ‘number of children regularly met’ as determinants of ‘finding a friend’ omits the fact that sending the child to kindergarten would, at least in our hypothetical example, only have an effect on the child finding a friend if there is no other intervention on whether it meets many other children.

Causal structure is also particularly relevant for the selection of control variables when trying to measure the effect size of a determinant, for instance in regression models. For example, for a person losing their job, having unemployment insurance will have a positive total effect on their life satisfaction compared to a counterfactual world without the insurance. A big part of this effect can be explained (is mediated) by the insurance payments allowing the person to maintain their standard of life (Fig. 1b). However, being insured is also likely to have an effect beyond the maintained standard of living, for instance, by sparing the individual from the potentially humiliating process of having to ask for social aid. In this example, it is clear that the insurance, the standard of living, and the avoided confrontation with the social welfare office are not at the same level of the causal chain. This means that neither standard of living nor meetings with the social welfare office should be selected as control variables if one is interested in the full effect of the insurance on life satisfaction. Consider a model regressing the life satisfaction of a freshly unemployed person on the existence of unemployment insurance. If one wants to assess the total effect of the insurance, controlling for the standard of living would lead to an underestimation of the effect and should therefore be refrained from. One would only control for the standard of living if one is interested in the remaining effect of the insurance beyond the fact that it allows one to maintain the standard of living. In any case, one should control for *confounders* (i. e., variables that influence both the dependent and independent variables, in this case well-being and the standard of living), such as pre-unemployment income^7^.

An inherent problem in well-being research is that conclusions can usually only be drawn from observational data, which limits causal claims. However, these limitations do not excuse researchers from thinking about causality and selecting control variables, based on the principles outlined above and their domain knowledge. Failing to do so (for instance, by simply controlling for any variable available) unnecessarily obscures the analysis.

The arguments on causal structure above, and especially the illustrations in Fig. 1, are implicitly based on causal inference theory in the tradition of Pearl (2000). While powerful, this causality framework has an important limitation: Usually, variables are either defined at one single point in time or have no notion of time (Peters et al., 2017, p. 10). A main assumption is acyclicity, i. e., that no variable can have a causal influence on itself, be it directly or via other variables. This is contrasted by the vast field of dynamical systems, in which the change over time of variables is explicitly modelled, allowing for feedback loops between system components. Most real-world systems, from biology to economics, are dynamically evolving and substantially driven by feedback loops (Meadows, 2008). This is also the case for the system of well-being determinants. For instance, a good relationship with a friend causes positive emotions, which then increase the motivation to take care of this friendship. Such feedback structures are typically represented by causal loop or stock-and-flow diagrams^8^. When interested in modelling the development of well-being and its determinants over time, it is usually better to use the dynamical systems approach. However, one can also often find a “static” formulation that allows using insights from causality theories based on the assumption of acyclity. The latter is the standard approach in most studies on well-being determinants. For instance, feedback loops do not have to be explicitly taken into account when studying the effect of educational interventions on life satisfaction a month after the intervention, or the effect of need satisfaction during childhood on skills as an adult, because the variables are defined at specific points in time. In both approaches, dynamic and static, it is crucial to reflect on a) the choice of considered time scales (for instance, some effects need time to proliferate) and b) the timing of interventions (as humans have a limited lifespan with specific phases).

We are not the first to point out the importance of causal structure in well-being research. For instance, Gough & Thomas (1994) highlighted the interconnectedness of factors influencing basic human needs satisfaction. Also, the concept of mediation will not come as a surprise to social scientists and psychologists trained in quantitative methods and has been used in studies on well-being (De Neve & Oswald, 2012; Diener & Tay, 2017; Diener et al., 2013; Godoy et al., 2006; Kaiser & Trinh, 2021; Newman et al., 2014; Reyes-García et al., 2016; Tay et al., 2018). However, both the presentation of well-being determinants and the development of quantitative models often ignore causal structure and interdependencies between determinants.

**Fig. 2:**
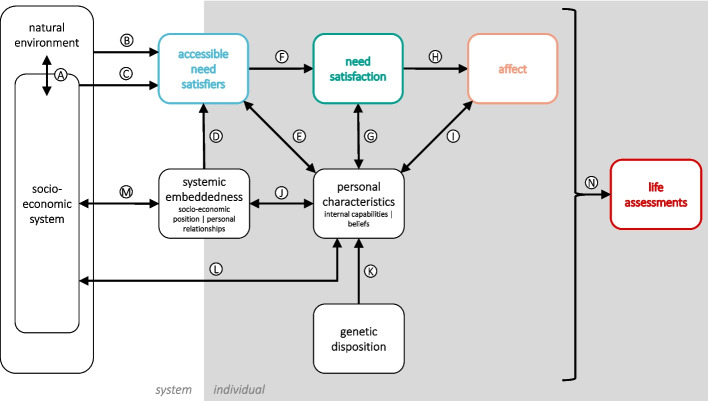
Framework of well-being determinants for one individual. Boxes represent categories of variables. The different background tones indicate whether the categories are located at the systemic or individual level, or at the interface between the two. In the case of systemic embeddedness and personal characteristics, there are two sub-categories. The term ‘internal capabilities’ (Nussbaum, 2011) summarizes concepts such as skills or personality. ‘Beliefs’ is an umbrella term for ideology, preferences, etc. (see Table 3 for more details). Arrows from one box to another depict direct causal links between the variables represented by the parent and child boxes. An arrow with two arrowheads means that there are causal links in both directions.

## The Framework

### Basic Principles

We propose a unified framework (Fig. 2) that addresses both the close interconnectedness of measures derived from different philosophical conceptions of well-being, as well as the need for a causal structure approach to their determinants. Our framework builds on several concepts developed in the well-being literature.

First of all, we differentiate between the influence of the environment a person lives in (as well as their position in it) and the influence of a person’s individual characteristics on their well-being. Veenhoven (2010) names these two categories *livability* and *life-ability*, respectively. In her capabilities approach, Nussbaum (2011) makes a very similar distinction between *external circumstances* and *internal capabilities*, where she defines internal capabilities as “characteristics of a person (personality traits, intellectual and emotional capacities, states of bodily fitness and health, internalized learning, skills of perception and movement)” (Nussbaum, 2011). This distinction has also been stressed in the well-being framework of the Centre for Well-being (Abdallah, 2011; Michaelson, 2014).

Secondly, we include the satisfaction of human needs (in the understanding of Max-Neef et al. (1991)) as a central pathway for the influence of any variable on affect and life assessments (Tamberg et al., 2024). We make a differentiation between needs, which are supposed to be universal, and satisfiers, which may vary between different socio-economic systems (Doyal & Gough, 1991; Max-Neef et al., 1991). According to Max-Neef et al. (1991), there are nine universal human needs: subsistence, protection, understanding, participation, leisure, creation, identity, and freedom.

Thirdly, we take into account that humans have the ability to perceive and evaluate all kinds of aspects of their lives, such as the general setup of the socio-economic system or their individual position in it, and not only their level of need satisfaction. These factors certainly influence life assessments *via their effect on need satisfaction*, but they may also be *directly evaluated* by this same person based on their values and expectations (which in turn are influenced by society). For instance, income might determine how well one can satisfy one’s needs in a market-based society, but it may also influence one’s life assessments because of the belief that a high income is something good in itself. Such an effect of income on life assessments beyond need satisfaction was, for instance, found by Diener et al. (1995); Tay and Diener (2011); Diener et al. (2010); Tamberg et al. (2024).

While our framework (Fig. 2) is motivated by the need for a causal perspective on the determinants of well-being (see section 3), it is not entirely consistent with the common graphical presentations of either causal networks (usually represented by directed acyclic graphs) or dynamical systems (usually represented by causal loop diagrams)^9^. Instead, it can be used as a template when developing a causal loop diagram or DAG for a specific research question involving concrete indicators.

### Variable Categories and Links

Graphically, our framework consists of boxes, which represent categories of variables (see Table 3), and arrows, which represent causal links between the variable categories (see Table 4). Given the conceptual nature of the framework, we do not make any assumption on the functional form of the dependencies (which can also differ between different variables represented by the same box). It may also involve the interaction of variables (i.e., the effect of one variable depends on the state of the other, and vice versa). Such an interaction can be described as a moderation of one variable’s effect by another. The dependency can also be such that the parent category defines the dimensions, types, or range of the variables in the child category. For instance, the socio-economic system defines the “landscape” of systemic embeddedness: In which dimensions socio-economic position manifests itself and the spectrum of possible positions (i.e., the shape of the distribution). In some cases, there is an overlap between the variables represented by two different boxes. Take, for instance, physical health: It is both an important component of the need for subsistence (Max-Neef et al., 1991) but also a personal characteristic that defines many abilities. In such a case, where a variable could be assigned to two categories depending on its role, we propose to read the causal link between the two boxes as an identity function.

While we summarise many variables in categories, these variables often have causal links between each other that are not discussed here. For instance, personality is known to impact ideological orientation (De Neve, 2015).

The graphical notation we use for causal relationships does not imply determinism but assumes stochastic influences. This is especially important in the case of the socio-economic position, which might, to a certain extent, depend on factors like personal skills, but which is nevertheless (at least in the current system) strongly determined by pure chance – most importantly, which family one was born into.

Since the focus of the framework is on the individual, feedback from the individual to the socio-economic system (link L) is weak for most individuals compared to the total effect of the socio-economic system on the individual via all potential pathways. The backwards link becomes important when looking at larger numbers of individuals, especially within social classes and politically oriented organisations, and their aggregated effect on society.

Many of the links we present contain human behaviour as an “implicit mediator”: For instance, the link between personal characteristics and personal relationships involves communication behaviours, based on one’s communication skills, that facilitate good relationships with other humans. The link between accessible need satisfiers and need satisfaction, which we portray in Table 4 as the decision of the individual to use a certain satisfier, also implicitly involves the behaviour of using the satisfier as another step in the causal connection. Given the ubiquity of the concept and the diversity of potential behaviours, we view behaviour as subsumed within the causal links.

One link that stands out is the one towards life assessments, as it comes from all other elements of the framework. When trying to form an overall life assessment, individuals potentially take into account their affective experience, their level of need satisfaction, the need satisfiers accessible to them, their systemic embeddedness, their personal characteristics, the social-economic system as a whole, as well as the natural environment it is part of. All these aspects are perceived and evaluated in a way that depends on personal characteristics such as values, preferences, expectations, and personality. The weighting of different aspects also depends on the exact question formulation (Nilsson et al., 2024).

**Table 3:** Variable categories with examples

Category	Examples
natural environment	climate and weather, biodiversity, natural resources
socio-economic system	political system, societal values, economic production, social movements, laws, culture ...
systemic embeddedness	
* socio-economic position*	relative income, level of power associated with job, privileges/discrimination
* personal relationships*	quantity and quality of relationships with friends, family members, romantic partners
personal characteristics	
* internal capabilities*	“personality traits, intellectual and emotional capacities, states of bodily fitness and health, internalized learning, skills of perception and movement” (Nussbaum, 2011), critical understanding, social skills
* beliefs*	ideological orientation, political opinions, personal values, preferences, expectations
accessible need satisfiers	work conditions, political rights, economic goods$^{a}$such as food, meetings with friends, festivities, clean air ...
need satisfaction	following Max-Neef et al. (1991): subsistence, protection, affection, understanding, participation, idleness, creation, identity, and freedom
affect	emotions and moods
life assessment	life satisfaction: “All things considered, how satisfied are you with your life as a whole these days? Use a 0 to 10 scale, where 0 is dissatisfied and 10 is satisfied.”,
	life evaluation (Cantril Ladder): “Please imagine a ladder with steps numbered from 0 at the bottom to 10 at the top. The top of the ladder represents the best possible life for you and the bottom of the ladder represents the worst possible life for you. On which step of the ladder would you say you personally feel you stand at this time?”

$^{a}$While Max-Neef makes an explicit differentiation between need satisfiers and economic goods, we argue that economic goods can be treated as a sub-category of need satisfiers without a loss of analytical power.

**Table 4:** Causal links with examples. The link directions correspond to Fig. 2

Link	Direction	Examples
A	$\downarrow$	• Climatic conditions influence crops and yields.
		• Reserves of fossil fuels enable a carbon-based economy.
	$\uparrow$	• Greenhouse gas emissions from economic activity change the global climate.
		• Mining activities, agriculture, and buildings alter entire landscapes.
B	$\rightarrow$	• Local climate conditions influence air quality.
C	$\rightarrow$	• The building stock influences housing availability.
		• For cycling to be accessible, the socio-economic system needs to provide adequate infrastructure.
		• The political system influences individuals’ opportunities for participation.
D	$\uparrow$	• In a money-based society, a higher relative income guarantees a higher share of economic outputs.
		• A position of esteem in society can serve as a satisfier for identity needs.
		• Personal relationships are satisfiers for needs such as affection and participation.
E	$\nwarrow$	• Knowing how to bike is a prerequisite for biking being an accessible need satisfier.
	$\searrow$	• Depending on the accessibility of cars and bikes and their associated infrastructures, a person will more strongly improve their ability to drive a car or to ride a bike.
		• In a car-dependent world, car-based social practices normalize everyday car use.
F	$\rightarrow$	• Biking can satisfy many different needs (subsistence, recreation, health, identity, ...).
G	$\uparrow$	• If biking is accessible, a person’s attitude towards it will influence whether they choose to bike or not.
	$\downarrow$	• Subsistence needs satisfaction leads to improved health and strength.
		• Lacking satisfaction of affection needs can lead to reduced social skills.
H	$\rightarrow$	• Food withdrawal leads to despair and anger.
		• The satisfaction of affection needs is usually accompanied by joy and relaxation.
I	$\nearrow$	• Some people tend to emotionally “swing” more than others in reaction to (missing) need satisfaction,
		• Different people have different mood “base levels”.
	$\swarrow$	• Feeling relaxed after exercising can motivate people to exercise more often.
		• Feelings of accomplishment in a learning situation help to remember the learned insight.
		• Pain motivates the avoidance of behaviours that caused it.
J	$\rightarrow$	• Meritocracy is believed more by members of upper than lower social classes.
		• Tastes in music, fashion, etc. differ by socio-economic group.
	$\leftarrow$	• Certain skills provide higher chances of acquiring a well-paid job.
		• People with social charisma often have more friends.
K	$\uparrow$	• Genes define the potential range of many personality traits, such as extraversion.
		• Some diseases, such as Huntington’s Chorea, are entirely genetically determined.
		• A genetically given talent for dancing can be activated by training.
L	$\rightarrow$	• A society with strong conservative family values will influence the individuals within it in that direction.
		• Socioeconomic systems shape what their members believe to be “normal human behaviour”.
	$\leftarrow$	• Individual skills define what can be produced in an economy (and how).
		• Using collective organising skills, individuals can influence the socio-economic system based on their political positions.
M	$\rightarrow$	• In a monetary-based socio-economic system, the socio-economic position will be linked to income.
		• Large inequalities within the socio-economic system will result in a large spectrum of possible individual socio-economic positions.
		• Family structures are influenced by marital rules.
	$\leftarrow$	• A powerful person’s beliefs have a stronger influence on the socio-economic system.
		• A person with many friends can more easily mobilise people for a cause they find important.

### The Roles of Income

**Fig. 3:**
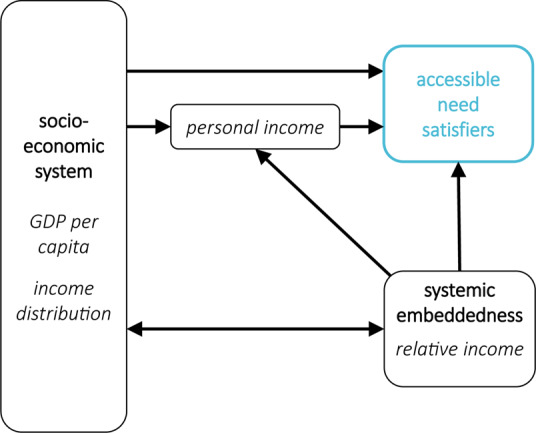
Zoom into the framework to illustrate the position of different income variables

Income is an important variable in many well-being studies (Diener et al., 1995; Easterlin, 1974; Easterlin & O’Connor, 2020; Kahneman & Deaton, 2010; Killingsworth, 2021; Killingsworth & Kahneman, 2023; Sacks et al., 2012). It can be differentiated into variables located at different places in the framework (see Fig. 3). First of all, there is a differentiation between income at the society level (usually measured as GDP per capita) and at the individual (or household) level. All else staying equal, people in richer countries have higher personal incomes, allowing them to buy more goods and services, which act as need satisfiers. This relationship is moderated by the income distribution and one’s position in it, i.e., one’s relative income (often measured as an income percentile). Beyond this effect via personal income, living in a richer country can also have a direct effect on accessible need satisfiers, for instance, by better public infrastructure.

### Situating Different Well-Being Philosophies

The different well-being philosophies presented in section 2 are interested in different variable categories represented in our framework. Most straightforward is the identification of hedonic well-being as affect.

For eudaimonic well-being in the objective list sense, either need satisfaction (human need theories) or the accessible need satisfiers (capabilities approach) are central. Eudaimonic well-being in the “flourishing” sense (i.e., in the SWB literature) is a bit more complex given the diversity of theories available. Following the classification proposed by Abdallah & Mahoney (2024), they can focus on personal characteristics, need satisfaction, or affect. Preference-based well-being can be understood as the accessible need satisfiers “weighted” by personal preferences (i.e., a subcategory of personal characteristics). As discussed in section 2, it is not clear which philosophical conception of well-being life assessments can be attributed to, or whether they should be seen as their own category.

## Application Examples

In the following, we will present examples of how to reason about causal effects on well-being using our framework. Given the great interest in well-being in sustainability research, after giving some examples of general societal interventions, we will focus on environmental impacts and how to decouple well-being from them (i.e., sustainable well-being).

### General Societal Interventions to Increase Human Well-Being

**Fig. 4:**
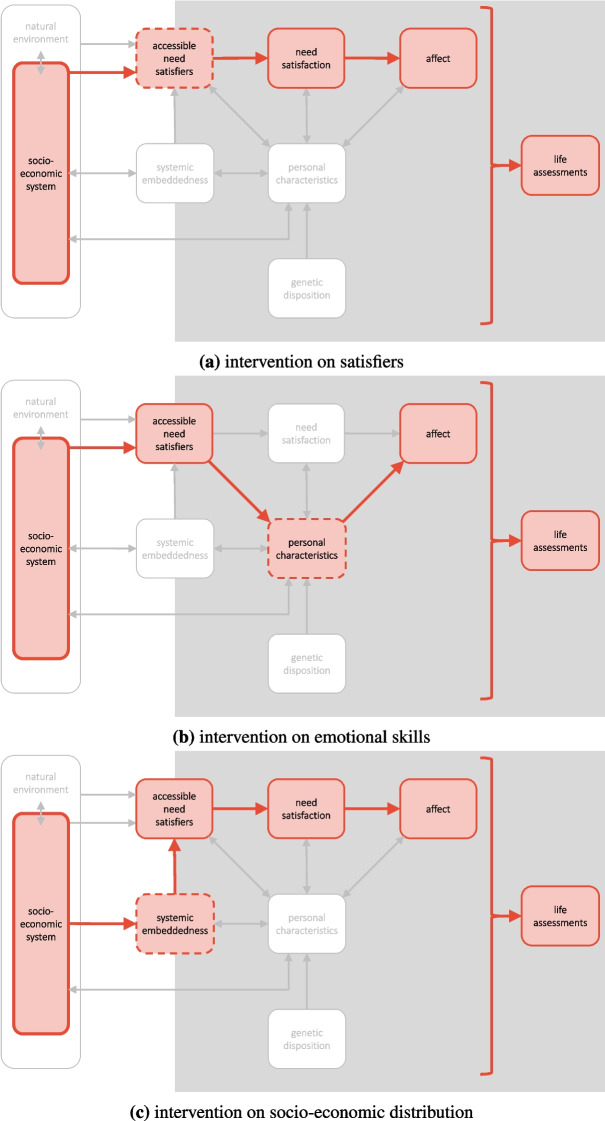
Examples of societal interventions on well-being

Societal interventions on well-being, while all stemming from changes in the socio-economic system, can target different determinants of well-being. Our framework helps to understand both the causal pathways that can be used to change these determinants and the potential downstream effects of such interventions. Here, we will discuss three example intervention types (Fig. 4).

First, a straightforward intervention target is the accessible need satisfiers (Fig. 4a). For instance, all else staying equal, increasing the accessibility of healthy food has a positive effect on need satisfaction (as well as on affect and life assessments further downstream) for an individual whose nutritional needs are not satisfied. This intervention type is not restricted to the production and distribution of goods and services. Changing aspects such as working conditions, decision/making processes, social rules, or information flows at the societal level can all change the quality of need satisfiers available to individuals.

Second, a society can also intervene in the emotional skills of their population, which are part of the category of ‘personal characteristics’ (Fig. 4b). For instance, it can increase the ability of its population to feel gratitude by integrating gratitude exercises in school curricula (implying a change in the need satisfiers accessible to school children). Gratitude interventions have been shown to have a significant positive effect on SWB indicators (Davis et al., 2016; Wood et al., 2010). In our framework, this could be explained by an increased “gratitude sensitivity” leading to a stronger affective reaction to need satisfaction (and possibly a less strong reaction to missing need satisfaction). Although this does not directly improve need satisfaction itself (which would be the relevant outcome for proponents of an objective lists interpretation of eudaimonia), it does improve hedonic well-being and likely also increases overall life assessments (via improved affect, but also via an influence of gratitude on the evaluation of one’s life).

Third, interventions in the socio-economic system can also change the importance of socio-economic position (which is part of systemic embeddedness) for well-being. Increasing the relative socio-economic position of one individual would, on the aggregated level, be cancelled out by the decreased position of others. However, what can be effectively intervened in at the societal level is the level of inequality. Human need satisfaction underlies strong saturation effects in relation to income (Tamberg et al., 2024). Therefore, decreasing inequality can (if the aggregate output is large enough) lift everyone above a necessary threshold to satisfy all human needs. This would lead to a better aggregate well-being outcome both in eudaimonic (on the needs level itself) and hedonic terms (because of the positive downstream effects of need satisfaction).

So far, the presentation of the examples above, as well as the illustrations, has been restricted to the first-order consequences on well-being, i.e., ignoring the dynamic feedbacks of the system. In principle, the analysis does not have to stop there. For example, it is clear that in the first case, improved satisfaction of nutritional needs can lead to improvements in different personal characteristics, such as physical skills, but also cognitive ability, with further positive downstream effects. In the gratitude exercise example, one can expect a feedback link from affect to personal characteristics. From our framework, it is not clear what the aggregate nature of this feedback will be. For example, improved affective balance (i.e., more positive affect and less negative affect) likely improves all kinds of psychological variables, which then have a positive downstream effect on need satisfaction via objective improvements in one’s life. At the same time, a high level of positive affect due to gratitude could reduce one’s drive to improve their situation individually and/or collectively, with a potential negative effect on need satisfaction compared to a person not gone through the intervention. This argument makes it clear that, in its current qualitative form, the framework helps to develop hypotheses about the potential causal effects of an intervention. However, it does not help to weigh competing long-term effects against each other when complex feedback loops are involved.

### Environmental Impacts on Well-Being

**Fig. 5:**
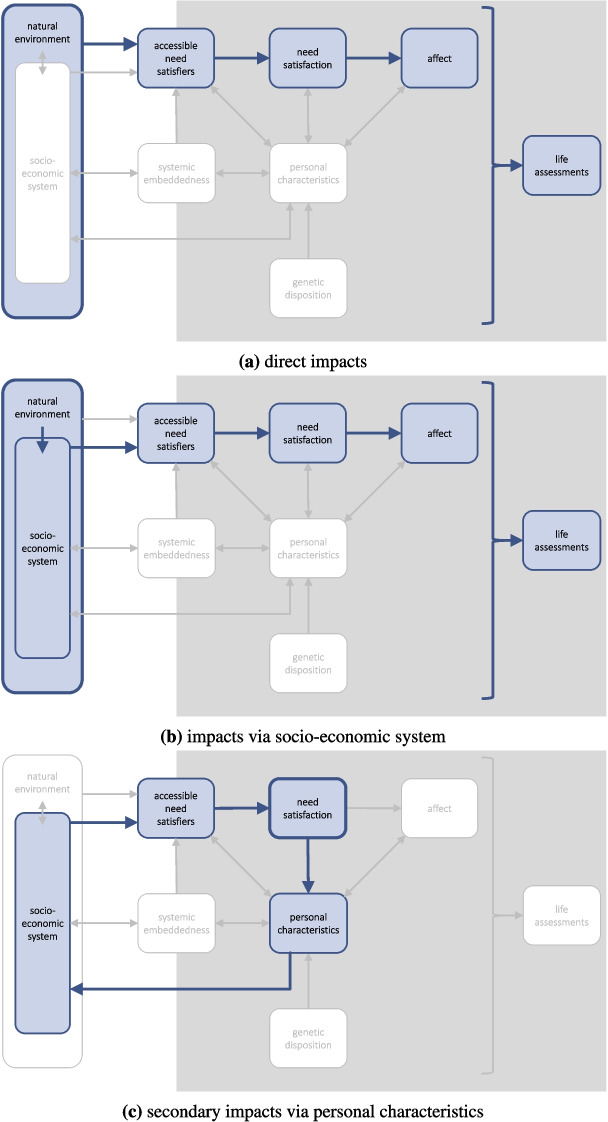
Illustration of climate impact types

As the system in which our economy and society are embedded, the environment influences human well-being in numerous ways. Given massive human interventions in the environment, such as climate change, it is important to take into account the pathways through which they impact human well-being (Adger et al., 2022). Our framework allows us to differentiate between three major pathways (Fig. 5):

- the direct link between the natural environment and need satisfiers, such as a flood destroying one’s house (potentially interacting with the ability of the society to protect houses from floods),
- indirect effects via the socio-economic system, such as the same flood destroying productive capacity and therefore endangering the provisioning of all kinds of need satisfiers, and
- additional feedback effects via changes of personal characteristics due to missing need satisfaction. For instance, an individual traumatised by a flood can contribute less to the societal provisioning of need satisfiers.

As in the case of societal interventions on well-being, further feedback effects beyond the direct impacts (as indicated in (c)) propagate through the system – in principle, infinitely so. For instance, reduced need satisfaction due to climate impacts can lead to apathy or increased social cohesion, leading to more or less climate action. For a proper assessment of the net long-term effects, a simple prosaic discussion of potential pathways is insufficient. Instead, a formal dynamic model would be needed, which comes with its own challenges in the context of complex social systems.

In Fig. 5, the effects of environmental change are considered assuming that initially all other variables remain equal. However, societal interventions can moderate these effects. For example, appropriate urban planning can make cities more resilient against heat waves or heavy rain (Dharmarathne et al., 2024; Keith & Meerow, 2022).

### Sustainable Well-Being

Environmental degradation negatively impacts human well-being in many ways. At the same time, economic activity correlates with ecological impacts. Therefore, a major question in sustainability science is how to ensure high levels of human well-being at low environmental damage. A typical answer in economics is technical efficiency. However, our framework illustrates two other main strategies:

- changing values, preferences, and skills (i.e., personal characteristics), allowing to satisfy human needs with less of the same or different satisfiers following sufficiency principles. This is especially true for social skills, as they directly improve the satisfaction of many needs and help to replace consumption-based satisfiers such as positional consumption. Changing satisfiers to reduce environmental impact often also has synergistic effects on well-being (Creutzig et al., 2022), such as in the case of directly and indirectly improved health due to more active mobility (Creutzig et al., 2012; Steg & Gifford, 2005; Woodcock et al., 2009).
- changing the shape of distributions determining socio-economic positions. The more equal the access to need satisfiers, the easier it is to lift everyone above necessary levels.

Following our framework, both arguments are straightforward for need satisfaction and affect. However, as discussed in section 2, it is not exactly clear which aspects individuals take into account when making an overall life assessment. Recent research shows that human need satisfaction explains most of the correlation between income and the Cantril Ladder life evaluation question (Tamberg et al., 2024). Given the strong correlation between income and ecological damage, this implies that a successful decoupling of human need satisfaction from income would also lead to a decoupling of life assessments from ecological damage.

## Conclusions

The framework proposed in this paper can be seen as an attempt to reconcile dominant well-being philosophies. We take an agnostic stance on the “correct” conceptualisation of well-being and the idea of “happiness maximization”, the latter being characteristic of Western, educated, industrial, rich, and democratic (“WEIRD”) societies (Krys et al., 2025). Instead, we show how the different conceptions of well-being cover different but closely connected categories in a dynamic causal network, and how variables intrinsic (or “ultimate” (Parfit, 1987)) to one well-being conception (i.e., the prudential good) are determinants of wellbeing (i.e., instrumental) for another. Some scholars argue that the philosophical debate on prudential versus instrumental goods can be distracting due to the close causal interconnectedness between the concepts in practice^10^. Instead, they argue in favour of a network theory of wellbeing (Bishop, 2015; Fabian, 2022), which requires a careful mapping of the causal network. Our framework can be used as a template for such a mapping, flexible enough to cover different societal settings.

In addition to the reconciliation of different well-being theories, the framework addresses the need for a causal structure perspective on the determinants of well-being. The application examples above illustrate how such a perspective helps to identify the well-being effects of societal interventions and environmental changes, as well as to reason about levers for sustainable well-being. They also show how, due to feedback links in the system, wellbeing is not just a consequence, but also a cause of societal processes – bringing a linear intervention perspective quickly to its limits.

While we discussed the examples mostly qualitatively, the framework can also be used as a basis for the development of quantitative models. For instance, there is a great interest in the Integrated Assessment Models community to extend climate mitigation scenarios to indicators of human well-being (Edwards et al., 2025; Funalot et al., 2025; Low et al., 2025; Wiedenhofer et al., 2024; Zimm et al., 2024). The main challenge for translating our framework into a model compatible with IAMs is the aggregation from the individual to the population level. Average levels of variables are often misleading as they hide deprivation in unequal societies. For instance, adequate levels of calorie intake can mask a strongly undernourished sub-population. Therefore, a proper tracking of distributions is important but computationally costly.

As discussed in Section 3, taking into account the causal structure of determinants is also important for a meaningful selection of variables in empirical studies, especially for regression models. Often, one wants to avoid controlling for the mediators of an effect (if one wants to estimate the full effect of an intervention, all else not being intervened on). However, some research questions require exactly this (if one wants to estimate the remaining effect of a variable beyond a known pathway, such as in Haberl et al. (2025); Tamberg et al. (2024)).

Mapping out the causal relationships of a complex system inevitably brings about two limitations. 

First, it will always lead to a simplification of reality, only depicting what are considered the most important variables and links. For our framework, this is even more the case as it summarises many variables in larger categories, and we focused on those variables that are most prominently discussed in the well-being literature. Therefore, some of the links are conceptually very “thick”. For instance, the links between affect and personal characteristics (I) represent complex phenomena studied in psychology, parts of the link from personal characteristics to the socio-economic system (L) are the area of interest of political science, and sociology researches, among other things, how the socio-economic system shapes the landscape of systemic embeddedness (M). As we showed for the case of income (Fig. 3), it is also possible to develop “zoom-ins” into parts of the framework that give a more detailed picture of the variables summarised in different categories. Such extensions of the causal diagrams could be especially useful for the very broad socio-economic system and environment part. For instance, one could integrate the energy service cascade (Kalt et al., 2019) for studies interested in the contribution of different energy services to well-being. A zoom-in that would be useful mostly for psychologists would entail a more explicit mental model of perception, affect, memory, anticipation, and evaluation, allowing a more detailed understanding of how hedonic well-being and life assessments form in the human psyche. This could, in principle, be combined with a more detailed account of time use to take into consideration the momentary nature of hedonic well-being (Han & Kaiser, 2024). Another important extension could address the various forms of human behaviour currently subsumed in many of the links.

Second, and related to that, mapping a system also means making a choice about the boundaries of that system. The focus of our framework is on one individual, for whom we take factors such as their genetic disposition as a given. By this, we exclude from the outset the dynamics that determine, for instance, the (epi)genetic dispositions in a population. The framework does include some important feedback links, such as from the personal characteristics of individuals to the socio-economic system^11^. However, it remains silent about the mechanisms translating individual values, opinions, and preferences to the societal level – such as collective organising, power structures, and opinion dynamics. Making these mechanisms more explicit could be a valuable extension of the framework.

Despite these limitations, which are inherent to modelling complex systems, we are convinced that our framework is useful for applications both in well-being and sustainability research, with potential topics ranging from education programs and workplace conditions to climate mitigation and economic redistribution. It can be used to define intervention points, anticipate their effects, and choose appropriate study designs to measure them – independent of the chosen well-being philosophy.

## Data Availability

The research did not use or generate any data.

## Footnotes

1 While there have been proposals for well-being frameworks in the literature, these existing ones typically only consist of theorised components of (subjective) well-being, not their determinants (White, 2010; Rauschmayer et al., 2010; Durayappah, 2011). Two frameworks come closer to what we are proposing in this paper. First, Juster et al. (1981) have proposed an economic accounting framework that can be understood as an implicit causal model. However, it is restricted to causal links from production systems towards households (without any feedbacks) and limited to an understanding of well-being as ‘utility’. Headey (1993) has adapted this accounting framework for the determinants of life satisfaction. Second, the Centre for Well-being of the new economics foundation uses a framework that makes a distinction between functionings and feelings on the well-being side as well as between external conditions and personal resources on the determinant side (Thompson & Marks, 2008; Abdallah, 2011; Michaelson, 2014).

2 Aristotle was not the only Ancient Greek philosopher concerned with the concept of ‘eudaimonia’ (which can in fact be translated as well-being). Rather, different streams in Ancient Greek philosophy proposed different definitions of the term. In this logic, Epicurean hedonism would also have to be understood as a eudaimonic theory, but one that equates eudaimonia with pleasure (Broadie, 2015). However, in the two well-being literature fields that we are focusing on here, eudaimonic well-being is usually understood as Aristotelian (Abdallah, 2011; Deci & Ryan, 2008; O’Neill, 2006). Throughout the article, we therefore use the term in this narrower interpretation.

3 The examples focus on the well-being of one individual, but could also be aggregated to the population level (e. g., by reporting the average blood pressure level, the percentage of undernourished people, the average reported intensity of positive feelings).

4 “Please imagine a ladder with steps numbered from 0 at the bottom to 10 at the top. The top of the ladder represents the best possible life for you and the bottom of the ladder represents the worst possible life for you. On which step of the ladder would you say you personally feel you stand at this time?”

5 “All things considered, how satisfied are you with your life as a whole these days? Use a 0 to 10 scale, where 0 is dissatisfied and 10 is satisfied.”

6 Clark et al. (2018) do present an ordering of variables into different time periods, which they also understand as a causal ordering. However, they do not present a full causal network taking into account influences inside of a time period, or additional confounders.

7 For a more detailed discussion of how to choose control variables (in causality called “adjustment sets”), see for instance (Peters et al., 2017).

8 Recently, there have been efforts in the causality field to extend its mathematical theories to the realm of dynamical systems (Blom et al., 2020; Bongers et al., 2022; Iwasaki & Simon, 1994; Mooij et al., 2013; Peters et al., 2022; Rubenstein et al., 2018). However, most contributions so far focus on the asymptotic behaviour of dynamical systems, which is not really helpful for the dynamics of human well-being.

9 Most evidently, the boxes in Fig. 2 represent categories of variables instead of single variables. Also, the framework includes links that lead to cycles. As discussed in section 3, this is normal in causal loop diagrams but not in DAGs.

10 Examples for nearly total correlations between different wellbeing concepts can be found in Goodman et al. (2018); Disabato et al. (2016); De Neve and Sachs (2020).

11 As discussed in section 3, feedback loops (i.e., cycles) are incompatible with classic causality theory and cannot be modelled with, for instance, regression models. Instead, they require complex system approaches, such as System Dynamics (Meadows, 2008). Whether or not this is necessary depends on the research question and the available methods.

## References

[CR1] Abdallah, S. (2011). *Measuring our progress: The power of well-being*. New Economics Foundation. https://new-economicsf.files.svdcdn.com/production/files/Measuring_our_Progress.pdf?dm=1555342865

[CR2] Abdallah, S., & Mahoney, J. (2024). Measuring eudaimonic components of subjective well-being: Updated evidence to inform national data collections. *OECD Papers on Well-being and Inequalities*. https://www.oecd.org/en/publications/measuring-eudaimonic-components-of-subjective-well-being_667fbe08-en.html.

[CR3] Adger, W. N., Barnett, J., Heath, S., & Jarillo, S. (2022). Climate change affects multiple dimensions of well-being through impacts, information and policy responses. *Nature Human Behaviour,**6*(11), 1465–1473. https://www.nature.com/articles/s41562-022-01467-8 10.1038/s41562-022-01467-8 36385175

[CR4] Aristotle (2014). *Nicomachean Ethics*. Translated by H. Rackham. Loeb Classical Library 73. Cambridge: Harvard University Press. https://www.loebclassics.com/view/LCL073/1926/volume.xml.

[CR5] Barrington-Leigh, C. (2024). The econometrics of happiness: Are we underestimating the returns to education and income? *Journal of Public Economics, **230*, 105052. https://www.sciencedirect.com/science/article/pii/S0047272723002347?via%3Dihub

[CR6] Benjamin, D. J., Cooper, K. B., Heffetz, O., Kimball, M. S., & Kundu, T. (2025). What do people want? *NBER Working Papers*. https://www.nber.org/papers/w33846

[CR7] Bishop, M. A. (2015). *The good life: Unifying the philosophy and psychology of well-being*. Oxford University Press.

[CR8] Blom, T., Bongers, S., & Mooij, J. M. (2020). Beyond Structural Causal Models: Causal Constraints Models. In *Proceedings of The 35th Uncertainty in Artificial Intelligence Conference*. https://proceedings.mlr.press/v115/blom20a.html.

[CR9] Bongers,S., Blom, T., & Mooij, J. M. (2022). Causal modeling of dynamical systems. *arXiv *[preprint]. https://arxiv.org/abs/1803.08784

[CR10] Brand-Correa, L. I., Mattioli, G., Lamb, W. F., & Steinberger, J. K. (2020). Understanding (and tackling) need satisfier escalation. *Sustainability: Science, Practice and Policy, **16*(1), 309–325. https://www.tandfonline.com/doi/full/10.1080/15487733.2020.1816026

[CR11] Brand-Correa, L. I., & Steinberger, J. K. (2017). A framework for decoupling human need satisfaction from energy use. *Ecological Economics*, *141*, 43–52. https://www.sciencedirect.com/science/article/pii/S0921800916308448?via%3Dihub

[CR12] Broadie, S. (2015). eudaimonism. In *Oxford Research Encyclopedia of Classics*. https://oxfordre.com/classics/display/10.1093/acrefore/9780199381135.001.0001/acrefore-9780199381135-e-7029.

[CR13] Clark, A., Flèche, S., Layard, R., Powdthavee, N., & Ward, G. (2018). *The origins of happiness: The science of well-being over the life course*. Princeton University Press. https://www.degruyter.com/document/doi/10.1515/9781400889129/html

[CR14] Creutzig, F., Mühlhoff, R., & Römer, J. (2012). Decarbonizing urban transport in European cities: four cases show possibly high co-benefits. *Environmental Research Letters,**7*(4), 044042. 10.1088/1748-9326/7/4/044042

[CR15] Creutzig, F., Niamir, L., Bai, X., Callaghan, M., Cullen, J., Díaz-José, J., Figueroa, M., Grubler, A., Lamb, W. F., Leip, A., Masanet, E., Mata, E., Mattauch, L., Minx, J. C., Mirasgedis, S., Mulugetta, Y., Nugroho, S. B., Pathak, M., Perkins, P., Joyashree, R., de la Rue, S., Saheb, Y., Some, S., Steg, L., Steinberger, J., & Ürge Vorsatz, D. (2022). Demand-side solutions to climate change mitigation consistent with high levels of well-being. *Nature Climate Change, 12*(1), 36–46. https://www.nature.com/articles/s41558-021-01219-y

[CR16] Crisp, R. (2021). Well-Being. In E. N. Zalta (Ed.) *The Stanford Encyclopedia of Philosophy*. Metaphysics Research Lab, Stanford University, winter 2021 ed. https://plato.stanford.edu/archives/win2021/entries/well-being/.

[CR17] Davis, D. E., Choe, E., Meyers, J., Wade, N., Varjas, K., Gifford, A., Quinn, A., Hook, J. N., Van Tongeren, D. R., Griffin, B. J., & Worthington, E. L., Jr. (2016). Thankful for the little things: A meta-analysis of gratitude interventions. *Journal of Counseling Psychology, **63*(1), 20–31. 10.1037/cou0000107 26575348

[CR18] De Neve, J.-E. (2015). Personality, Childhood Experience, and Political Ideology. *Political Psychology,**36*(1), 55–73. https://onlinelibrary.wiley.com/doi/pdf/10.1111/pops.12075 10.1111/pops.12075 PMC6867614 31749510

[CR19] De Neve, J.-E., & Oswald, A. J. (2012). Estimating the influence of life satisfaction and positive affect on later income using sibling fixed effects. *Proceedings of the National Academy of Sciences, **109*(49), 19953–19958. https://www.pnas.org/doi/abs/10.1073/pnas.1211437109 10.1073/pnas.1211437109 PMC3523830 23169627

[CR20] De Neve, J.-E., & Sachs, J. D. (2020). The SDGs and human well-being: A global analysis of synergies, trade-offs, and regional differences. *Scientific Reports,**10*(1), 15113. https://www.nature.com/articles/s41598-020-71916-9. 10.1038/s41598-020-71916-9 PMC7492223 32934290

[CR21] Deci, E. L., & Ryan, R. M. (2008). Hedonia, eudaimonia, and well-being: An introduction. *Journal of Happiness Studies,**9*(1), 1–11. 10.1007/s10902-006-9018-1

[CR22] Deeming, C. (2013). Addressing the Social Determinants of Subjective Wellbeing: The Latest Challenge for Social Policy. *Journal of Social Policy, **42*(3), 541–565. https://www.cambridge.org/core/journals/journal-of-social-policy/article/addressing-the-social-determinants-of-subjective-wellbeing-the-latest-challenge-for-social-policy/D0FC22F52A6912F7A0A1E923D7B19D19. 10.1017/S0047279413000202 PMC3663082 23710101

[CR23] Dharmarathne, G., Waduge, A. O., Bogahawaththa, M., Rathnayake, U., & Meddage, D. P. P. (2024). Adapting cities to the surge: A comprehensive review of climate-induced urban flooding. *Results in Engineering, **22*, 102123. https://www.sciencedirect.com/science/article/pii/S2590123024003773.

[CR24] Diener, E. (1984). Subjective well-being. *Psychological Bulletin*, *95*(3), 542–575. 6399758

[CR25] Diener, E., Diener, M., & Diener, C. (1995). Factors predicting the subjective well-being of nations. *Journal of Personality and Social Psychology*, *69*, 851–864. https://psycnet.apa.org/fulltext/1996-11062-001.html 7473035 10.1037//0022-3514.69.5.851

[CR26] Diener, E., Ng, W., Harter, J., & Arora, R. (2010). Wealth and happiness across the world: Material prosperity predicts life evaluation, whereas psychosocial prosperity predicts positive feeling. *Journal of Personality and Social Psychology, **99*(1), 52–61. 10.1037/a0018066. 10.1037/a0018066 20565185

[CR27] Diener, E., Oishi, S., & Tay, L. (2018). Advances in subjective well-being research. *Nature Human Behaviour*, *2*(4), 253–260. https://www.nature.com/articles/s41562-018-0307-6 10.1038/s41562-018-0307-6 30936533

[CR28] Diener, E., Oishi, S., Tay, L., & Zhu, Z. (2018b). *Handbook of Well-Being*. DEF Publishers. https://digitalcommons.unomaha.edu/psychfacbooks/9.

[CR29] Diener, E., & Tay, L. (2017). A scientific review of the remarkable benefits of happiness for successful and healthy living. *Happiness Transforming the Development Landscape,**6*, 90–117. https://philpapers.org/archive/ADLDOT.pdf#page=95.

[CR30] Diener, E., Tay, L., & Oishi, S. (2013). Rising income and the subjective well-being of nations. *Journal of Personality and Social Psychology*, *104*(2), 267–276. https://psycnet.apa.org/doiLanding?doi=10.1037%2Fa0030487 23106249 10.1037/a0030487

[CR31] Dietz, T. (2023). *Decisions for sustainability: Facts and values*. Cambridge University Press, (1 ed). https://www.cambridge.org/core/product/identifier/9781009169400/type/book.

[CR32] Disabato, D. J., Goodman, F. R., Kashdan, T. B., Short, J. L., & Jarden, A. (2016). Different types of well-being? A cross-cultural examination of hedonic and eudaimonic well-being. *Psychological Assessment, **28*(5), 471–482. 10.1037/pas0000209 26348031

[CR33] Dodds, S. (1997). Towards a ‘science of sustainability’: Improving the way ecological economics understands human well-being. *Ecological Economics, **23*(2), 95–111. https://www.sciencedirect.com/science/article/pii/S0921800997000475.

[CR34] Dolan, P., Peasgood, T., Dixon, A., Knight, M., Phillips, D., Tsuchiya, A., & White, M. (2006). *Research on the relationship between well-being and suatinable development*. Report for Defra. https://eprints.chi.ac.uk/id/eprint/1168/1/WellbeingProject3A.pdf

[CR35] Dolan, P., Peasgood, T., & White, M. (2008). Do we really know what makes us happy? A review of the economic literature on the factors associated with subjective well-being. *Journal of Economic Psychology, **29*(1), 94–122. https://www.sciencedirect.com/science/article/pii/S0167487007000694.

[CR36] Doyal, L., & Gough, I. (1984). A theory of human needs. *Critical Social Policy, **4*(10), 6–38. 10.1177/026101838400401002.

[CR37] Doyal, L., & Gough, I. (1991). *A theory of human need*. Basingstoke: Macmillan. OCLC: 59832551.

[CR38] Driver, J. (2022). The History of Utilitarianism. In E. N. Zalta, & U. Nodelman (Eds.) *The Stanford Encyclopedia of Philosophy*. Metaphysics Research Lab, Stanford University, winter 2022 ed. https://plato.stanford.edu/archives/win2022/entries/utilitarianism-history/.

[CR39] Durayappah, A. (2011). The 3P model: A general theory of subjective well-being. *Journal of Happiness Studies,**12*(4), 681–716. 10.1007/s10902-010-9223-9

[CR40] Easterlin, R. A. (1974). Does economic growth improve the human lot? Some empirical evidence. In P. A. David, & M. W. Reder (Eds.) *Nations and households in economic growth* (pp. 89–125). Academic Press. https://www.sciencedirect.com/science/article/pii/B9780122050503500087.

[CR41] Easterlin, R. A., & O’Connor, K. J. (2020). The Easterlin paradox. In K. F. Zimmermann (Ed.) *Handbook of labor, human resources and population economics* (pp. 1–25). Cham: Springer International Publishing. https://link.springer.com/10.1007/978-3-319-57365-6_184-1.

[CR42] Edwards, A., Brockway, P. E., Bickerstaff, K., & Nijsse, F. J. M. M. (2025). Towards modelling post-growth climate futures: A review of current modelling practices and next steps. *Environmental Research Letters,**20*(5), 053005. 10.1088/1748-9326/adc9c6

[CR43] Fabian, M. (2022). *A Theory of Subjective Wellbeing. *Oxford University Press.

[CR44] Fleurbaey, M. (2009). Beyond GDP: The quest for a measure of social welfare. *Journal of Economic Literature,**47*(4), 1029–1075. https://pubs.aeaweb.org/doi/10.1257/jel.47.4.1029

[CR45] Funalot, P., Jean, M. S., & Rougier, E. (2025). *Transitioning towards harmonious living: A society-economy-nature model with heterogeneous agents, finite resources and politics (SEN-HARP) for Europe-27*. https://hal.science/hal-05005292v1/file/BSE_SPES_2025_Funalot.pdf

[CR46] Gasper, D. (2007). Human well-being: Concepts and conceptualizations. In M. McGillivray (Ed.) *Human Well-Being* (pp. 23–64). London: Palgrave Macmillan UK. http://link.springer.com/10.1057/9780230625600_2.

[CR47] Godoy, R. A., Reyes-García, V., McDade, T., Huanca, T., Leonard, W. R., Tanner, S., & Vadez, V. (2006). Does village inequality in modern income harm the psyche? Anger, fear, sadness, and alcohol consumption in a pre-industrial society. *Social Science & Medicine, **63*(2), 359–372. https://www.sciencedirect.com/science/article/pii/S0277953606000499. 10.1016/j.socscimed.2006.01.021 16519979

[CR48] Goodman, F. R., Disabato, D. J., Kashdan, T. B., & Kauffman, S. B. (2018). Measuring well-being: A comparison of subjective well-being and PERMA. *The Journal of Positive Psychology, **13*(4), 321–332. 10.1080/17439760.2017.1388434

[CR49] Gough, I., & Thomas, T. (1994). Why do levels of human welfare vary among nations? *International Journal of Health Services,**24*(4), 715–748. 10.2190/KHAM-M986-W67T-56B7 7896471 10.2190/KHAM-M986-W67T-56B7

[CR50] Haberl, H., Duro, J., Grammer, B., Perez-Laborda, A., Brockway, P., Heun, M., Pachauri, S., Reiter, C., Striessnig, E., Krausmann, F., Streeck, J., & Wiedenhofer, D. (2025). Towards sustainable provision of services supporting many years of good life: A global panel analysis 1990–2019. *Research Square* [preprint]. https://www.researchsquare.com/article/rs-6228143/v1.

[CR51] Han, J., & Kaiser, C. (2024). Time use and happiness: US evidence across three decades. *Journal of Population Economics,**37*(1), 15. 10.1007/s00148-024-00982-4

[CR52] Haybron, D. (2020). Happiness. In E. N. Zalta (Ed.) *The Stanford Encyclopedia of Philosophy*. Metaphysics Research Lab, Stanford University, summer 2020 ed. https://plato.stanford.edu/archives/sum2020/entries/happiness/.

[CR53] Headey, B. (1993). An economic model of subjective well-being: Integrating economic and psychological theories. *Social Indicators Research,**28*(2), 97–116. 10.1007/BF01079653

[CR54] Helliwell, J., Layard, R., & Sacha, J. (2015). *World Happiness Report 2015*. Tech. rep., Sustainable Development Solutions Network, New York. https://worldhappiness.report/ed/2015/.

[CR55] Honderich, T. (2005). *The Oxford companion to philosophy*. OUP Oxford.

[CR56] Huppert, F. A. (2009). Psychological well-being: Evidence regarding its causes and consequences. *Applied Psychology: Health and Well-Being, **1*(2), 137–164. https://onlinelibrary.wiley.com/doi/abs/10.1111/j.1758-0854.2009.01008.x.

[CR57] Iwasaki, Y., & Simon, H. A. (1994). Causality and model abstraction. *Artificial Intelligence, **67*(1), 143–194. https://www.sciencedirect.com/science/article/pii/0004370294900140.

[CR58] Juster, F. T., Courant, P. N., & Dow, G. K. (1981). A theoretical framework for the measurement of well-being. *Review of Income and Wealth, **27*(1), 1–31. https://onlinelibrary.wiley.com/doi/pdf/10.1111/j.1475-4991.1981.tb00190.x

[CR59] Kahneman, D., & Deaton, A. (2010). High income improves evaluation of life but not emotional well-being. *Proceedings of the National Academy of Sciences, **107*(38), 16489–16493. https://www.pnas.org/doi/abs/10.1073/pnas.1011492107. 10.1073/pnas.1011492107 PMC2944762 20823223

[CR60] Kahneman, D., & Krueger, A. B. (2006). Developments in the measurement of subjective well-Being. *Journal of Economic Perspectives, **20*(1), 3–24. https://pubs.aeaweb.org/doi/pdfplus/10.1257/089533006776526030

[CR61] Kaiser, C., & Trinh, N. A. (2021). Positional, mobility, and reference effects: How does social class affect life satisfaction in europe? *European Sociological Review,**37*(5), 713–730. 10.1093/esr/jcaa067

[CR62] Kalt, G., Wiedenhofer, D., Görg, C., & Haberl, H. (2019). Conceptualizing energy services: A review of energy and well-being along the Energy Service Cascade. *Energy Research & Social Science, **53*, 47–58. https://www.sciencedirect.com/science/article/pii/S2214629618311757.

[CR63] Keith, L., & Meerow, S. (2022). *Planning for urban heat resilience*. American Planning Association. https://repository.arizona.edu/handle/10150/664007

[CR64] Killingsworth, M. A. (2021). Experienced well-being rises with income, even above $75,000 per year. *Proceedings of the National Academy of Sciences, **118*(4), e2016976118. https://www.pnas.org/doi/10.1073/pnas.2016976118. 10.1073/pnas.2016976118 PMC7848527 33468644

[CR65] Killingsworth, M. A., Kahneman, D., & Mellers, B. (2023). Income and emotional well-being: A conflict resolved. *Proceedings of the National Academy of Sciences*, *120*(10), e2208661120. https://www.pnas.org/doi/abs/10.1073/pnas.2208661120 10.1073/pnas.2208661120 PMC10013834 36857342

[CR66] Krys, K., Kostoula, O., van Tilburg, W. A. P., Mosca, O., Lee, J. H., Maricchiolo, F., Kosiarczyk, A., Kocimska-Bortnowska, A., Torres, C., Hitokoto, H., Liew, K., Bond, M. H., Lun, V.M.-C., Vignoles, V. L., Zelenski, J. M., Haas, B. W., Park, J., Vauclair, C.-M., Kwiatkowska, A., … Uchida, Y. (2025). Happiness maximization is a WEIRD way of living. *Perspectives on Psychological Science,**20*(5), 874–902. 10.1177/17456916231208367 38350096 10.1177/17456916231208367 PMC12408936

[CR67] Lamb, W. F., & Steinberger, J. K. (2017). Human well-being and climate change mitigation. *WIREs Climate Change, **8*(6), e485. https://onlinelibrary.wiley.com/doi/abs/10.1002/wcc.485.

[CR68] Layard, R., & De Neve, J.-E. (2023). *Wellbeing: Science and policy*. Cambridge University Press, 1 ed. https://www.cambridge.org/core/product/identifier/9781009298957/type/book.

[CR69] Low, S., Brutschin, E., Baum, C. M., & Sovacool, B. K. (2025). Expert perspectives on incorporating justice considerations into integrated assessment modelling. *NPJ Climate Action, **4*(1), 1–12. https://www.nature.com/articles/s44168-025-00218-5.

[CR70] Max-Neef, M. A., Elizalde, A., & Hopenhayn, M. (1991). *Human scale development: Conception, application and further reflections*. New York: The Apex Press.

[CR71] Meadows, D. H. (2008). *Thinking in systems: A primer. *Chelsea Green Publishing.

[CR72] Michaelson, J. (2014). Practical models for well-being-oriented policy. In T. J. Hämäläinen, & J. Michaelson (Eds.) *Well-Being and beyond*. Edward Elgar Publishing. https://china.elgaronline.com/view/edcoll/9781783472895/9781783472895.00024.xml.

[CR73] Mitsis, P. (1988). Pleasure, happiness, and desire. In *Epicurus’ ethical theory: The pleasures of invulnerability* (vol. 48, pp. 11–58). Cornell University Press. https://www.jstor.org/stable/10.7591/j.cttq45fk.5.

[CR74] Mooij, J. M., Janzing, D., & Schölkopf, B. (2013). From Ordinary Differential Equations to Structural Causal Models: The deterministic case. *arXiv* [preprint]. http://arxiv.org/abs/1304.7920.

[CR75] Newman, D. B., Tay, L., & Diener, E. (2014). Leisure and subjective well-being: A model of psychological mechanisms as mediating factors. *Journal of Happiness Studies,**15*(3), 555–578. 10.1007/s10902-013-9435-x

[CR76] Nilsson, A. H., Eichstaedt, J. C., Lomas, T., Schwartz, A., & Kjell, O. (2024). The Cantril Ladder elicits thoughts about power and wealth. *Scientific Reports,**14*(1), 2642. https://www.nature.com/articles/s41598-024-52939-y. 10.1038/s41598-024-52939-y PMC10834405 38302578

[CR77] Nussbaum, M. C. (2000). *Women and human development: The capabilities approach*. Cambridge University Press.

[CR78] Nussbaum, M. C. (2011). *Creating capabilities: The human development approach*. Belknap Press, Harvard University Press.

[CR79] Nussbaum, M. C. (2015). Philosophy and economics in the capabilities approach: An essential dialogue. *Journal of Human Development and Capabilities,**16*(1), 1–14. 10.1080/19452829.2014.983890

[CR80] Nussbaum, M. C., & Sen, A. (1993). *The quality of life*. Oxford University Press.

[CR81] O’Neill, J. (2006). Citizenship, well-being and sustainability: Epicurus or Aristotle? *Analyse & Kritik, **28*(2), 158–172. https://www.degruyter.com/document/doi/10.1515/auk-2006-0203/html.

[CR82] O’Neill, J. (2010). The overshadowing of need. In F. Rauschmayer, I. Omann, & J. Frühmann (Eds.), *Sustainable development: Capabilities, needs, and well-being. *Routledge.

[CR83] Parfit, D. (1987). *Reasons and persons*. Oxford: Clarendon Press

[CR84] Pearl, J. (2000). *Causality Models, reasoning, and inference*. Cambridge, U.K.; New York : Cambridge University Press. http://archive.org/details/causalitymodelsr0000pear.

[CR85] Peters, J., Bauer, S., & Pfister, N. (2022). Causal models for dynamical systems*. *In* Probabilistic and Causal Inference: The Works of Judea Pearl* (vol. 36, pp. 671–690). New York: Association for Computing Machinery, 1 ed. 10.1145/3501714.3501752

[CR86] Peters, J., Janzing, D., & Schölkopf, B. (2017). *Elements of causal inference: Foundations and learning algorithms*. The MIT Press. https://library.oapen.org/handle/20.500.12657/26040

[CR87] Rauschmayer, F., Omann, I., & Frühmann, J. (2010). Needs, capabilities and quality of life: Refocusing sustainable development. In Sustainable development: Capabilities, needs, and well-being. https://www.routledge.com/Sustainable-Development-Capabilities-Needs-and-Well-being/Rauschmayer-Omann-Fruhmann/p/book/9780415516815

[CR88] Reyes-García, V., Babigumira, R., Pyhälä, A., Wunder, S., Zorondo-Rodríguez, F., & Angelsen, A. (2016). Subjective Wellbeing and Income: Empirical Patterns in the Rural Developing World. *Journal of Happiness Studies,**17*(2), 773–791. 10.1007/s10902-014-9608-2 27642259 10.1007/s10902-014-9608-2 PMC5023045

[CR89] Rogers, D. S., Duraiappah, A. K., Antons, D. C., Munoz, P., Bai, X., Fragkias, M., & Gutscher, H. (2012). A vision for human well-being: Transition to social sustainability. *Current Opinion in Environmental Sustainability, **4*(1), 61–73. https://www.sciencedirect.com/science/article/pii/S1877343512000140.

[CR90] Rubenstein, P. K., Bongers, S., Schoelkopf, B., & Mooij, J. M. (2018). From Deterministic ODEs to Dynamic Structural Causal Models*. arXiv *[preprint]*.* http://arxiv.org/abs/1608.08028.

[CR91] Ryan, R. M., & Deci, E. L. (2000). Intrinsic and extrinsic motivations: Classic definitions and new directions. *Contemporary Educational Psychology, **25*(1), 54–67. https://www.sciencedirect.com/science/article/pii/S0361476X99910202. 10.1006/ceps.1999.1020 10620381

[CR92] Ryan, R. M., & Deci, E. L. (2001). On happiness and human potentials: A review of research on hedonic and eudaimonic Well-Being. *Annual Review of Psychology,**52*(1), 141–166. 10.1146/annurev.psych.52.1.141 10.1146/annurev.psych.52.1.141 11148302

[CR93] Ryff, C. D. (1989). Happiness is everything, or is it? Explorations on the meaning of psychological well-being. *Journal of Personality and Social Psychology, **57*(6), 1069–1081. https://psycnet.apa.org/fulltext/1990-12288-001.html

[CR94] Sacks, D. W., Stevenson, B., & Wolfers, J. (2012). The new stylized facts about income and subjective well-being. *Emotion*, *12*(6), 1181. https://psycnet.apa.org/fulltext/2012-32386-001.pdf 23231724 10.1037/a0029873

[CR95] Schrijver, I., Behrens, P., Hoekstra, R., & Kleijn, R. (2025). Inclusion of wellbeing impacts of climate change: A review of literature and integrated environment–society–economy models. *The Lancet Planetary Health*, 9(12). https://pubmed.ncbi.nlm.nih.gov/41317742/10.1016/j.lanplh.2025.101375 41317742

[CR96] Sen, A. (2008). The economics of happiness and capability. In *Capabilities and happiness*, vol. 27. Oxford University Press, Oxford.

[CR97] Steg, L., & Gifford, R. (2005). Sustainable transportation and quality of life. *Journal of Transport Geography, **13*(1), 59–69. https://www.sciencedirect.com/science/article/pii/S0966692304000870.

[CR98] Tamberg, L., Steinberger, J., Cologna, V., Fisch-Romito, V., Millward-Hopkins, J., Calvo, F., & Oreskes, N. (2024). Human need satisfaction enables decoupling of well-being from income. *Research Square* [preprint]. https://www.researchsquare.com/article/rs-5355955/v1.

[CR99] Tay, L., & Diener, E. (2011). Needs and subjective well-being around the world. *Journal of Personality and Social Psychology*, *101*, 354–365. https://psycnet.apa.org/buy/2011-12249-001 21688922 10.1037/a0023779

[CR100] Tay, L., & Kuykendall, L. (2013). Promoting happiness: The malleability of individual and societal subjective wellbeing. *International Journal of Psychology,**48*(3), 159–176. 10.1080/00207594.2013.779379 23551025 10.1080/00207594.2013.779379

[CR101] Tay, L., Pawelski, J. O., & Keith, M. G. (2018). The role of the arts and humanities in human flourishing: A conceptual model. *The Journal of Positive Psychology, **13*(3), 215–225. https://www.tandfonline.com/doi/full/10.1080/17439760.2017.1279207.

[CR102] Thompson, S., & Marks, N. (2008). *Measuring well-being in policy: Issues and applications*. New Economics Foundation: Tech. rep.

[CR103] Veenhoven, R. (2010). Greater happiness for a greater number: Is that possible and desirable? *Journal of Happiness Studies,**11*, 605–629. https://www.researchgate.net/publication/254758162_Greater_Happiness_for_a_Greater_Number_Is_that_Possible_and_Desirable

[CR104] Vittersø, J. (2025). *Humanistic Wellbeing: Toward a Value-Based Science of the Good Life*. Cham: Springer Nature Switzerland. https://link.springer.com/10.1007/978-3-031-69292-5.

[CR105] Weizsäcker, C. C. v. (2024). *Freedom and Adaptive Preferences*. London: Routledge.

[CR106] White, S. C. (2010). Analysing wellbeing: A framework for development practice. *Development in Practice,**20*(2), 158–172. 10.1080/09614520903564199

[CR107] Wiedenhofer, D., Streeck, J., Wiese, F., Verdolini, E., Mastrucci, A., Ju, Y., Boza-Kiss, B., Min, J., Norman, J., Wieland, H., Bento, N., León, M. F. G., Magalar, L., Mayer, A., Gingrich, S., Hayashi, A., Jupesta, J., Ünlü, G., Niamir, L., & Pauliuk, S. (2024). Industry transformations for high service provisioning with lower energy and material demand: A review of models and scenarios. *Annual Review of Environment and Resources*, *49*(49), 249–279. https://www.annualreviews.org/content/journals/10.1146/annurev-environ-110822-044428

[CR108] Wood, A. M., Froh, J. J., & Geraghty, A. W. A. (2010). Gratitude and well-being: A review and theoretical integration. *Clinical Psychology Review, **30*(7), 890–905. https://www.sciencedirect.com/science/article/pii/S0272735810000450. 10.1016/j.cpr.2010.03.005 20451313

[CR109] Woodcock, J., Edwards, P., Tonne, C., Armstrong, B. G., Ashiru, O., Banister, D., Beevers, S., Chalabi, Z., Chowdhury, Z., Cohen, A., Franco, O. H., Haines, A., Hickman, R., Lindsay, G., Mittal, I., Mohan, D., Tiwari, G., Woodward, A., & Roberts, I. (2009). Public health benefits of strategies to reduce greenhouse-gas emissions: Urban land transport. *The Lancet, **374*(9705), 1930–1943. 10.1016/S0140-6736(09)61714-1 19942277

[CR110] Zimm, C, Mintz-Woo, K., Brutschin, E., Hanger-Kopp, S., Hoffmann, R., Kikstra, J. S., Kuhn, M., Min, J., Muttarak, R., Pachauri, S., Patange, O., Riahi, K., & Schinko, T. (2024). Justice considerations in climate research. *Nature Climate Change*, *14*(1), 22–30. https://www.nature.com/articles/s41558-023-01869-0.

